# Commentary: Use of acupuncture in stroke and stroke complications: a systematic review and meta-analysis based on sham-controlled trials

**DOI:** 10.3389/fneur.2026.1750747

**Published:** 2026-02-18

**Authors:** Runcheng Wang, Yibin Zhao, Lihua Xuan

**Affiliations:** 1 The First School of Clinical Medicine, Zhejiang Chinese Medical University, Hangzhou, Zhejiang, China; 2 Department of Acupuncture and Moxibustion, The First Affiliated Hospital of Zhejiang Chinese Medical University (Zhejiang Provincial Hospital of Chinese Medicine), Hangzhou, Zhejiang, China

**Keywords:** acupuncture, commentary, meta-anaiysis, sham-controlled trial, stroke

We have read with interest the paper by Wang et al., titled “Use of acupuncture in stroke and stroke complications: a systematic review and meta-analysis based on sham-controlled trials,” recently published in *Frontiers in Neurology* (1). This study, based on 24 randomized controlled trials (RCTs) involving 2,310 patients, suggested that acupuncture was a promising treatment for stroke and stroke complications, as it was shown to significantly improve the quality of life, neurological function, and depressive symptoms of patients in comparison to sham acupuncture. We appreciate the authors' contributions to this field; however, we have some suggestions for improvement.

First, the literature search, which covered seven electronic databases, identified 24 included trials, 23 of which originated from China and only one from the Republic of Korea, indicating a notable geographic bias. According to Jüni et al., evidence derived from single-country trials carries a higher risk of bias toward exaggerated benefits and has limited external validity (2). In addition, the majority of the included trials were single-center studies with total sample sizes below 100, suggesting that the pooled outcomes may be inflated due to small-study effects (SSE), which can result from publication bias (3). Consequently, the above risks of bias represent a major limitation that casts doubt on the validity of the overall findings.

Second, the included studies involved different stroke types, such as cerebral infarction and intracerebral hemorrhage, as well as various acupuncture therapies and disease courses, introducing considerable heterogeneity into the pooled outcomes (4). According to Okuda et al., researchers should consider different stroke types when developing clinical treatment strategies for stroke patients, as patients with intracerebral hemorrhage require a longer recovery period than those with cerebral infarction (5). Furthermore, the authors exhibited a critical oversight in the standardization of intervention dosage. A clear example of this is the difference between Boyang et al. (6), who employed a continuous wave of 2 Hz, and Ma et al. (7), who utilized a dilatational wave of 2/15 Hz. A study by Yao et al. found that using a lower frequency of electroacupuncture can more effectively improve motor function after focal cerebral infarction in rats, as evidenced by the shortened latent period and increased wave amplitude of motor evoked potentials (8). The limitations above constitute a major source of heterogeneity, urging caution in interpreting the pooled results and diminishing confidence in the findings.

Finally, the majority of the included trials lacked follow-up after treatment. Stroke is a common and serious disease that can lead to long-term disability; hence, long-term follow-up is essential for evaluating the efficacy of acupuncture (9). However, among the 24 included RCTs, only three trials conducted follow-up over 6 months after treatment and reported the clinical outcomes. Thus, the evidence based on these studies was insufficient to evaluate the long-term efficacy of acupuncture in stroke and its complications.

In summary, although limited by certain methodological issues, the authors' work nonetheless offers a comprehensive evaluation to date of the efficacy and safety of acupuncture treatment in stroke and stroke complications. We hope that future studies will build upon this foundational work by using more rigorous methodologies to validate and extend these findings.
